# Comparison of the mutation patterns between tumor tissue and cell-free DNA in stage IV gastric cancer

**DOI:** 10.18632/aging.204512

**Published:** 2023-02-10

**Authors:** Ching-Yun Kung, Wen-Liang Fang, Yi-Ping Hung, Kuo-Hung Huang, Ming-Huang Chen, Yee Chao, Shih-Chieh Lin, Anna Fen-Yau Li, Su-Shun Lo, Chew-Wun Wu

**Affiliations:** 1 Department of Surgery, Division of General Surgery, Taipei Veterans General Hospital, Taipei, Taiwan; 2 School of Medicine, National Yang Ming Chiao Tung University, Taipei, Taiwan; 3 Department of Oncology, Center of Immuno-Oncology, Taipei Veterans General Hospital, Taipei, Taiwan; 4 Department of Pathology, Taipei Veterans General Hospital, Taipei, Taiwan; 5 Department of Anatomical Pathology, Cheng Hsin General Hospital, Taipei, Taiwan; 6 National Yang Ming Chiao Tung University Hospital, Yilan, Taiwan

**Keywords:** tumor DNA, cfDNA, NGS, sensitivity, specificity

## Abstract

Compared to stage I–III gastric cancer (GC), the level of cell-free DNA (cfDNA) was significantly higher in stage IV GC. The mutation patterns of different metastatic patterns between cfDNA and tumor DNA in stage IV GC have not yet been reported. We used next-generation sequencing (NGS) to analyze cfDNA and tumor DNA in 56 stage IV GC patients. Tumor DNA and cfDNA were analyzed using a 29-gene NGS panel. In tumor samples, the most commonly mutated gene was *TP53* (64%), followed by *ARID1A* (62%), *KMT2C* (60%) and *KMT2D* (58%). In cfDNA samples, the most commonly mutated genes were *FAT4* (19%) and *MACF1* (19%), followed by *KMT2D* (18%), *ARID1A* (14%) and *LRP1B* (14%). The concordance of mutation patterns in these 29 genes was 42.0% between cfDNA and tumor DNA. A specificity of 100% was found when using the mutation status of cfDNA to predict mutations in tumor samples. The sensitivity of the mutation status of cfDNA to predict mutation in tumor samples was highest in *FAT4* (88.9%), followed by *MACF1* (80%), *CDH1* (75%) and *PLB1* (75%). For cfDNA with *PLB1* mutations, patients were more likely to develop distant lymphatic metastasis than peritoneal metastasis. Patients with multiple-site metastases had significantly more mutated spots than patients with single-site metastasis. Due to the high sensitivity and specificity of some genes in the prediction of mutation in tumor samples, monitoring the mutation pattern of cfDNA may be useful in the stage IV GC treatment.

## INTRODUCTION

Gastric cancer (GC) is the sixth most common cancer and the second most common cause of cancer-related death worldwide [1]. Radical gastrectomy with lymph node dissection remains the mainstay of GC treatment with curative intent. However, some GC patients were diagnosed at a late stage even stage IV disease, and most of them died within one year. The metastatic patterns of GC include peritoneal, hematogenous and distant lymphatic metastases. However, small metastatic peritoneal nodules without ascites formation, tiny liver metastases and borderline-sized paraaortic lymph nodes may be regarded as the absence of distant metastasis by abdominal computed tomography scan. In addition, tumor markers, carcinoembryonic antigen (CEA) and carbohydrate antigen 19-9 (CA 19-9), are even within the normal range in some stage IV GC patients.

Circulating cell-free DNA (cfDNA) can serve as a liquid biopsy and noninvasive method for cancer monitoring and management [2–4]. cfDNA levels in GC patients are more sensitive than CEA levels in the prediction of tumor recurrence [5, 6], and the cfDNA levels are significantly higher in stage IV GC [5].

Next-generation sequencing (NGS) has been used to investigate the concordance of genetic mutations between tumor tissue DNA and cfDNA [7]. Although high concordance of genetic mutation patterns was reported between tumor tissue DNA and cfDNA, some mutant variants were found in tumor tissue DNA only, and some were found in cfDNA only. The discrepancy in genetic mutations between primary tumor DNA and cfDNA was considered to be due to tumor heterogeneity. For stage I–III esophagus cancer and GC, a recent NGS study [8] with enrollment of 295 patients demonstrated that the cfDNA was detected in 96% preoperatively and 23.5% within 16 weeks after surgery. Their results showed that cfDNA detected at any time point after surgery was associated with shorter recurrence-free survival and poor prognosis. In addition, genetic methylation in cfDNA was reported to be a biomarker for predicting lymphatic metastasis, distant metastasis, peritoneal metastasis, and patient survival in GC [9–13]. However, the correlation between genetic mutations and metastatic patterns in tumor DNA and cfDNA in stage IV GC is still unknown.

In the current study, we used NGS and evaluated the concordance of genetic mutations between tumor DNA and cfDNA in stage IV GC patients. The relationship between the genetic mutations and metastatic patterns was analyzed.

## RESULTS

### Clinicopathological characteristics

The clinicopathological features of the 56 stage IV GC patients are shown in Table 1. There were 29 males and 27 females. The mean age was 65.8 years old. The tumor was located mostly in the middle third of the stomach. Approximately 69.6% of the 56 patients had poorly differentiated tumors and 92.9% had lymphovascular invasion. The most common metastatic site was the peritoneum, followed by distant lymphatic and hematogenous metastases. Among the 56 stage IV GC patients, five patients had metastases in both the peritoneum and distant lymph nodes; one patient had metastases in both the peritoneum and hematogenous metastases.

**Table 1:** Clinicopathological features of 56 stage IV gastric cancer (GC) patients.

**Clinicopathological features**	**Stage IV GC (*n* = 56) (%)**
Age	65.8 ± 13.9
Gender (M/F)	29/27
Tumor size (cm)	7.8 ± 2.9
Tumor location	
Upper third	6 (10.7)
Middle third	23 (41.1)
Lower third	21 (37.5)
Whole stomach	6 (10.7)
Cell differentiation	
Well/moderate	17 (30.4)
Poor	39 (69.6)
Lymphovascular invasion	52 (92.9)
Lauren’s classification	
Intestinal type	19 (33.9)
Diffuse type	37 (66.1)
Metastatic site	
Hematogenous	3 (5.4)
Peritoneum	33 (58.9)
Distant lymph node	26 (46.4)

### Analysis of the mutated genes in GC tumor samples and cfDNA

Among the 56 stage IV GC patients, genetic mutations were observed in both tumor DNA and cfDNA in 54 (96.4%) patients. Two patients had no genetic mutations detected in either tumor DNA or cfDNA.

**Figure 1:**
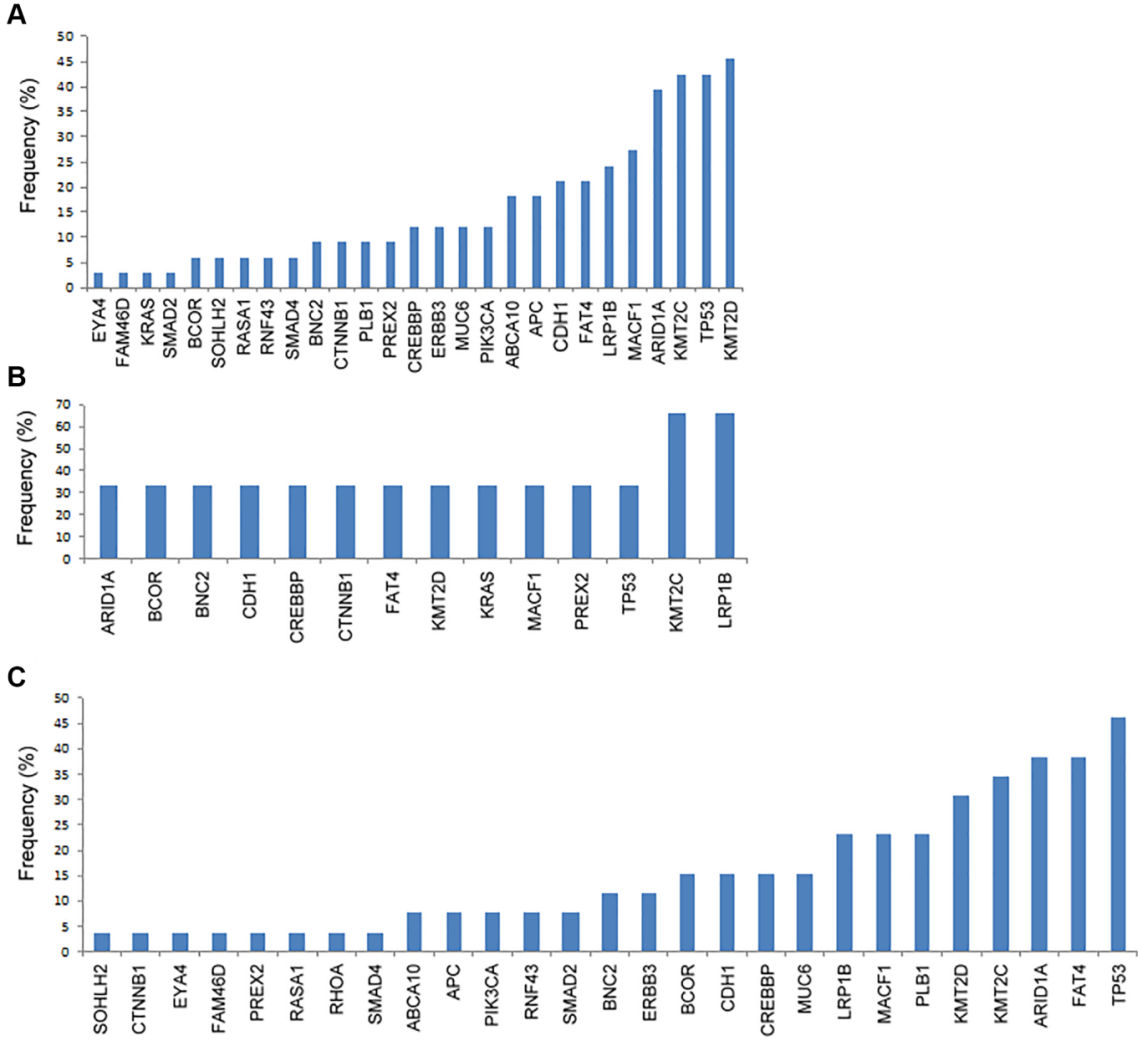
**The frequency of genetic mutations according to the metastatic patterns.** (**A**) Peritoneal metastasis, (**B**) hematogenous metastasis, and (**C**) distant lymphatic metastasis.

We analyzed the genetic mutation according to the metastatic sites. As shown in Figure 1A, for patients with peritoneal metastasis, the most commonly mutated gene was *KMT2D* (45%), followed by *TP53* (42%), *KMT2C* (42%), *ARID1A* (39%), *MACF1* (27%), *LRP1B* (24%), *FAT4* (21%), *CDH1* (21%), *APC* (18%), and *ABCA10* (18%). In Figure 1B, for patients with hematogenous metastasis, the most commonly mutated genes were *LRP1B* (67%) and *KMT2C* (67%), followed by *TP53* (33%), *PREX2* (33%), *MACF1* (33%), *KRAS* (33%), *KMT2D* (33%), *FAT4* (33%), *CTNNB1* (33%), *CREBBP* (33%), *CDH1* (33%), *BNC2* (33%), *BCOR* (33%), and *ARID1A* (33%). In Figure 1C, for patients with distant lymphatic metastasis, the most commonly mutated gene was *TP53* (46%), followed by *FAT4* (38%), *ARID1A* (38%), *KMT2C* (35%), *PLB1* (31%), *MACF1* (23%), *LRP1B* (23%), *MUC6* (23%), *CREBBP* (15%), *CDH1* (15%), *BCOR* (15%), *ERBB3* (15%), and *BNC2* (15%). The most common mutated spot in both peritoneal and distant lymphatic metastases was KMT2C c.8390delA.

**Figure 2:**
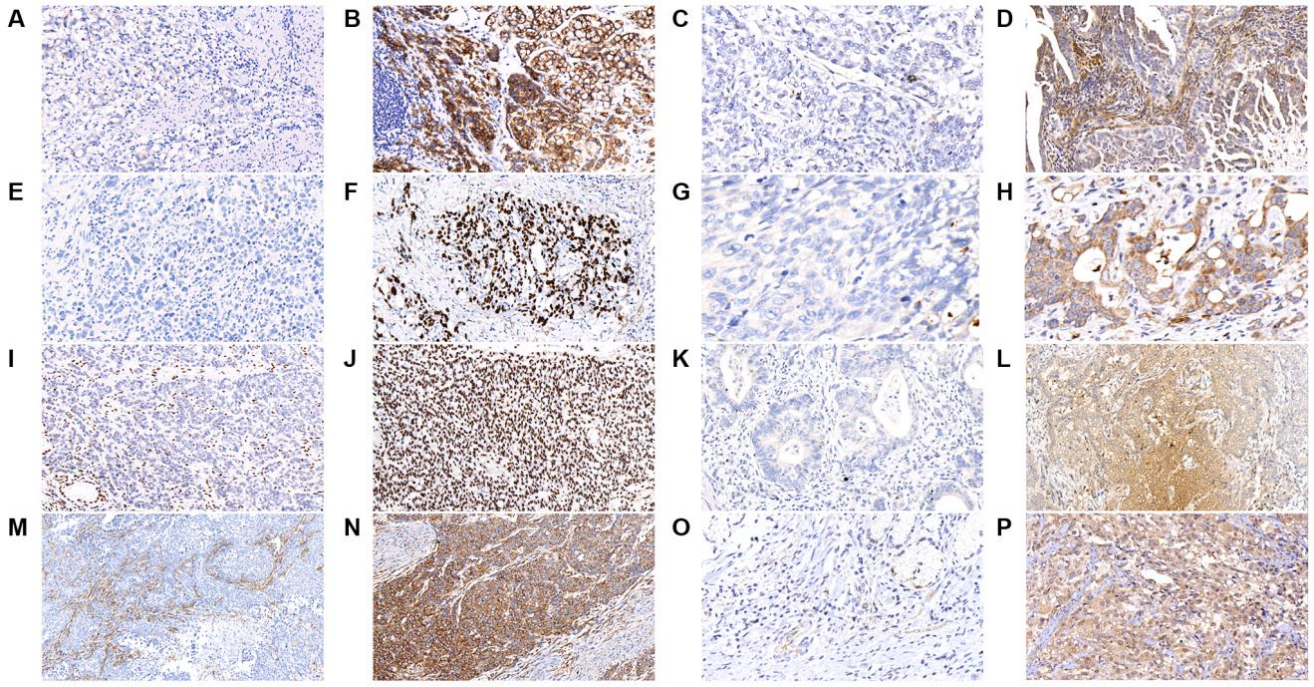
**The results of immunohistochemical staining in gastric cancer tumor samples are shown as follows in the figures.** (**A**) E-cadherin-negative (**B**) E-cadherin-positive (**C**) MACF1-negative (**D**) MACF1-positive (**E**) p53-negative (**F**) p53-positive (**G**) PLB1-negative (**H**) PLB1-positive (**I**) ARID1A-negative (**J**) ARID1A-positive (**K**) KMT2C-negative (**L**) KMT2C-positive (**M**) FAT4-negative (**N**) FAT4-positive (**O**) KMT2D-negative (**P**) KMT2D-positive. (x400).

We further analyzed the immunohistochemical (IHC) stain for some common mutated genes in the tumor samples, including *CDH1*, *MACF1*, *TP53*, *PLB1*, *ARID1A*, *KMT2C*, *FAT4*, and *KMT2D*. As shown in Figure 2 and Table 2, tumors with *CDH1* mutations were associated with significantly more negative expression of the correlated E-cadherin protein (*P* = 0.021), which was also observed in tumors with mutations in *MACF1* (*P* < 0.001), *TP53* (*P* < 0.001), *PLB1* (*P* < 0.001), *ARID1A* (*P* < 0.001), *KMT2C* (*P* < 0.001), *FAT4* (*P* < 0.001), and *KMT2D* (*P* < 0.001).

**Table 2:** The correlation between the expression of IHC stain and genetic mutation.

**IHC stain**	**Genetic mutation**		***P* value**
	**No *n* (%)**	**Yes *n* (%)**	
E-cadherin (CDH1)			0.021
Negative	0	1 (11.1)	
Positive	47 (100)	8 (88.9)	
MACF1			<0.001
Negative	0	7 (58.3)	
Positive	44 (100)	5 (41.7)	
P53			<0.001
Negative	3 (8.8)	14 (63.6)	
Positive	31 (91.2)	8 (36.4)	
PLB1			<0.001
Negative	1 (2.1)	8 (100)	
Positive	47 (97.9)	0	
ARID1A			<0.001
Negative	1 (2.8)	12 (60.0)	
Positive	35 (97.2)	8 (40.0)	
KMT2C			<0.001
Negative	1 (2.4)	11 (73.3)	
Positive	40 (97.6)	4 (26.7)	
FAT4			<0.001
Negative	2 (4.7)	6 (46.2)	
Positive	41 (95.3)	7 (53.8)	
KMT2D			<0.001
Negative	0	8 (44.4)	
Positive	38 (100)	10 (55.6)	

Abbreviation: IHC: immunohistochemical.

**Figure 3:**
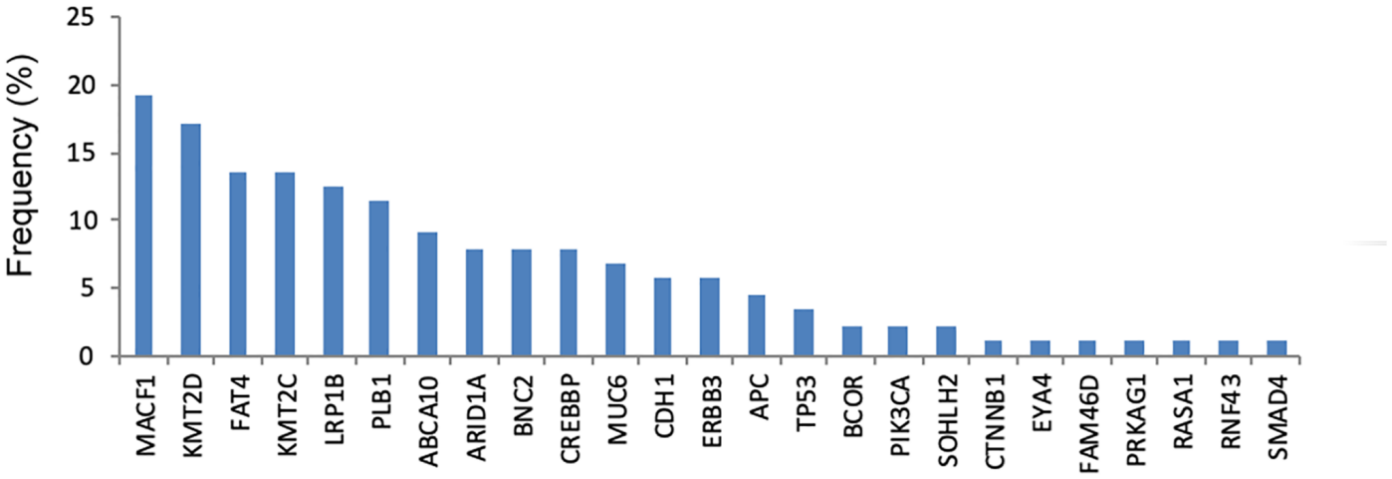
The frequency of genetic mutations in cfDNA.

As shown in Figure 3, the most commonly mutated gene in cfDNA was *MACF1* (19.3%), followed by *KMT2D* (17.0%), *FAT4* (13.6%), *KMT2C* (13.6%), *LRP1B* (12.5%) and *PLB1* (11.4%). The median number of mutated genes in the 56 GC cfDNA samples was 1 (range 0–5). Among them, twenty-six (46.4%) patients had more than one mutated gene.

**Figure 4:**
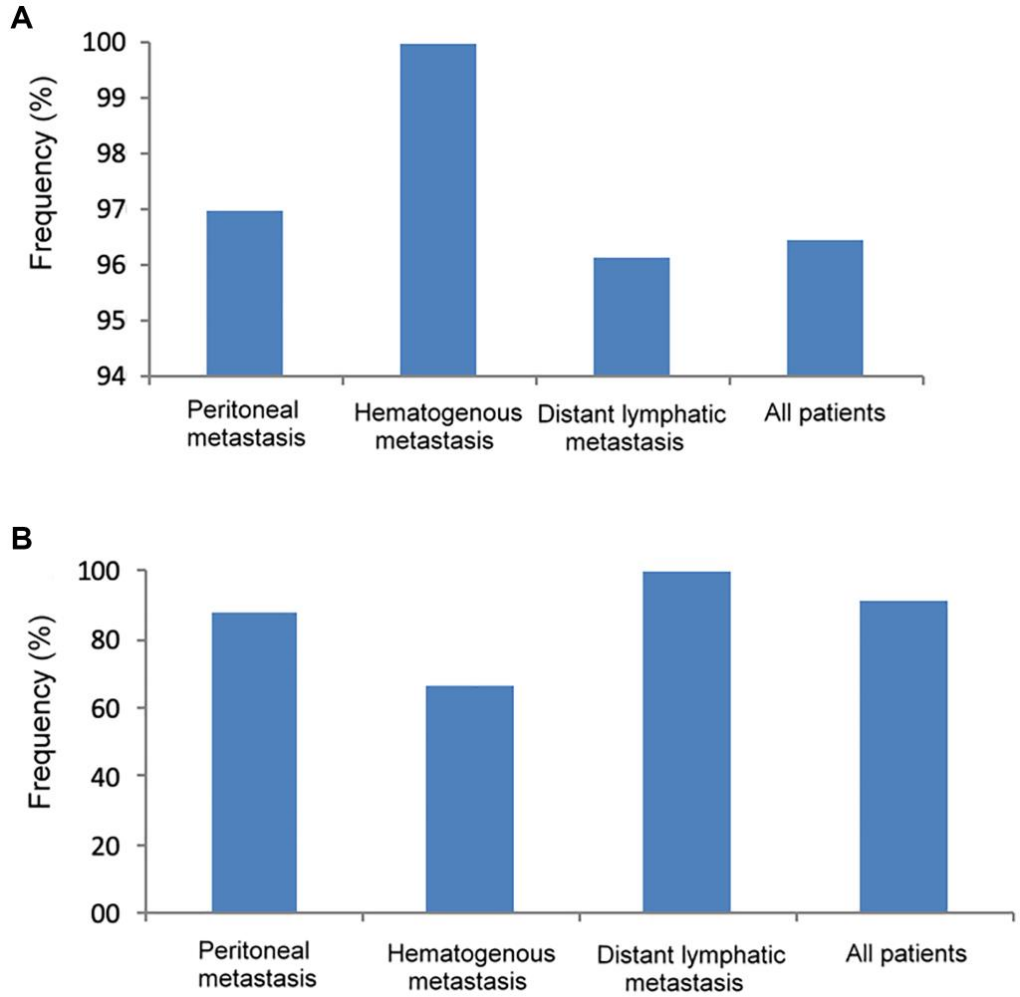
**The frequency of genetic mutations in tumor DNA and cfDNA according to different metastatic patterns.** (**A**) Tumor DNA, (**B**) cfDNA.

As shown in Figure 4A, the frequency of genetic mutations (at least one gene mutation) in tumor DNA was highest in patients with hematogenous metastasis (100%), followed by peritoneal metastasis (97%) and distant lymphatic metastasis (96.2%). In Figure 4B, the frequency of genetic mutation in cfDNA was highest in patients with distant lymphatic metastasis (100%), followed by peritoneal metastasis (87.5%) and hematogenous metastasis (66.7%).

As shown in Table 3, comparing patients with peritoneal metastasis alone and distant lymphatic metastasis alone, there was no significant difference in the frequency of any of the genetic mutations between these two metastatic patterns in tumor DNA; however, in cfDNA, the frequency of *PLB1* mutations was significantly higher in patients with distant lymphatic metastasis than in those with peritoneal metastasis (23.8% vs. 3.7%, *P* = 0.037).

**Table 3:** The correlation of tumor DNA mutation, cfDNA mutation, single peritoneal metastasis and single distant lymphatic metastasis.

**Genetic mutation**	**Tumor DNA**			**cfDNA**		
	**Peritoneum *n* = 27 *n* (%)**	**Distant LM *n* = 21 *n* (%)**	***P* value**	**Peritoneum *n* = 27 *n* (%)**	**Distant LM *n* = 21 *n* (%)**	***P* value**
*CREBBP*	2 (7.4)	2 (9.5)	0.792	1 (3.7)	1 (4.8)	0.856
*TP53*	11 (40.7)	8 (38.1)	0.853	3 (11.1)	0	0.115
*ARID1A*	11 (40.7)	7 (33.3)	0.599	4 (14.8)	3 (14.3)	0.959
*BCOR*	1 (3.7)	4 (19.0)	0.084	0	1 (4.8)	0.252
*CTNNB1*	1 (3.7)	0	0.373	0	0	
*KMT2D*	10 (37.0)	6 (28.6)	0.537	8 (29.6)	2 (9.5)	0.089
*RNF43*	1 (3.7)	1 (4.8)	0.856	0	0	
*APC*	3 (11.1)	0	0.115	2 (7.4)	0	0.203
*FAT4*	3 (11.1)	7 (33.3)	0.060	2 (7.4)	6 (28.6)	0.051
*KMT2C*	8 (29.6)	6 (28.6)	0.936	4 (14.8)	2 (9.5)	0.582
*ABCA10*	5 (18.5)	2 (9.5)	0.381	5 (18.5)	2 (9.5)	0.381
*MUC6*	2 (7.4)	2 (9.5)	0.792	1 (3.7)	2 (9.5)	0.409
*KRAS*	0	0		0	0	
*SMAD4*	1 (3.7)	0	0.373	1 (3.7)	0	0.373
*PIK3CA*	3 (11.1)	1 (4.8)	0.430	0	0	
*BNC2*	2 (7.4)	2 (9.5)	0.792	1 (3.7)	2 (9.5)	0.409
*PLB1*	3 (11.1)	5 (23.8)	0.242	1 (3.7)	5 (23.8)	**0.037**
*MACF1*	7 (25.9)	4 (19.0)	0.574	5 (18.5)	3 (14.3)	0.696
*ERBB3*	4 (14.8)	3 (14.3)	0.959	2 (7.4)	2 (9.5)	0.792
*CDH1*	4 (14.8)	2 (9.5)	0.582	3 (11.1)	2 (9.5)	0.858
*RASA1*	2 (7.4)	1 (4.8)	0.707	1 (3.7)	0	0.373
*PREX2*	2 (7.4)	1 (4.8)	0.707	0	0	
*EYA4*	1 (3.7)	1 (4.8)	0.856	1 (3.7)	0	0.373
*LRP18*	6 (22.2)	5 (23.8)	0.897	3 (11.1)	4 (19.0)	0.440
*FAM46D*	1 (3.7)	1 (4.8)	0.856	0	1 (4.8)	0.252
*SMAD2*	1 (3.7)	2 (9.5)	0.409	0	0	
*RHOA*	0	1 (4.8)	0.252	0	0	
*SOHLH2*	2 (7.4)	1 (4.8)	0.707	1 (3.7)	1 (4.8)	0.856
*CNGA4*	0	0		0	0	

Abbreviations: cfDNA: cell-free DNA; LM: lymphatic metastasis.

The specificity of all genetic mutations in cfDNA was 100% in predicting mutations in tumor DNA. As shown in Table 4, the sensitivity of the top nine mutated genes in tumor DNA and cfDNA was compared. Using the mutation pattern of cfDNA in the prediction of mutations in tumor DNA, the sensitivity was the highest in *FAT4* (88.9%), followed by *MACF1* (80%), *CDH1* (75%), *PLB1* (75%), *KMT2D* (72.7%), *LRP1B* (71.4%), *KMT2C* (40%), *ARID1A* (25%), and *TP53* (13.6%).

**Table 4:** Sensitivity and specificity of nine top mutated genes between tumor DNA and cfDNA of stage IV GC patients.

	***TP53***		***ARID1A***		***KMT2C***		***KMT2D***		***FAT4***		***LRP1B***		***MACF1***		***CDH1***		***PLB1***	
	**Tumor DNA mutation**		**Tumor DNA mutation**		**Tumor DNA mutation**		**Tumor DNA mutation**		**Tumor DNA mutation**		**Tumor DNA mutation**		**Tumor DNA mutation**		**Tumor DNA mutation**		**Tumor DNA mutation**	
	**Yes**	**No**	**Yes**	**Yes**	**Yes**	**No**	**Yes**	**No**	**Yes**	**No**	**Yes**	**No**	**Yes**	**No**	**Yes**	**No**	**Yes**	**No**
cfDNA mutation																		
Yes	3	0	5	0	6	0	8	0	8	0	5	0	4	0	3	0	3	0
No	19	34	15	36	9	41	3	45	1	47	2	49	1	51	1	52	1	52
Sensitivity	13.6%		25%		40%		72.7%		88.9%		71.4%		80%		75%		75%	
Specificity	100%		100%		100%		100%		100%		100%		100%		100%		100%	

Abbreviations: cfDNA: cell-free DNA; GC: gastric cancer.

### Concordance of genetic mutations between cfDNA and tumor samples

**Figure 5:**
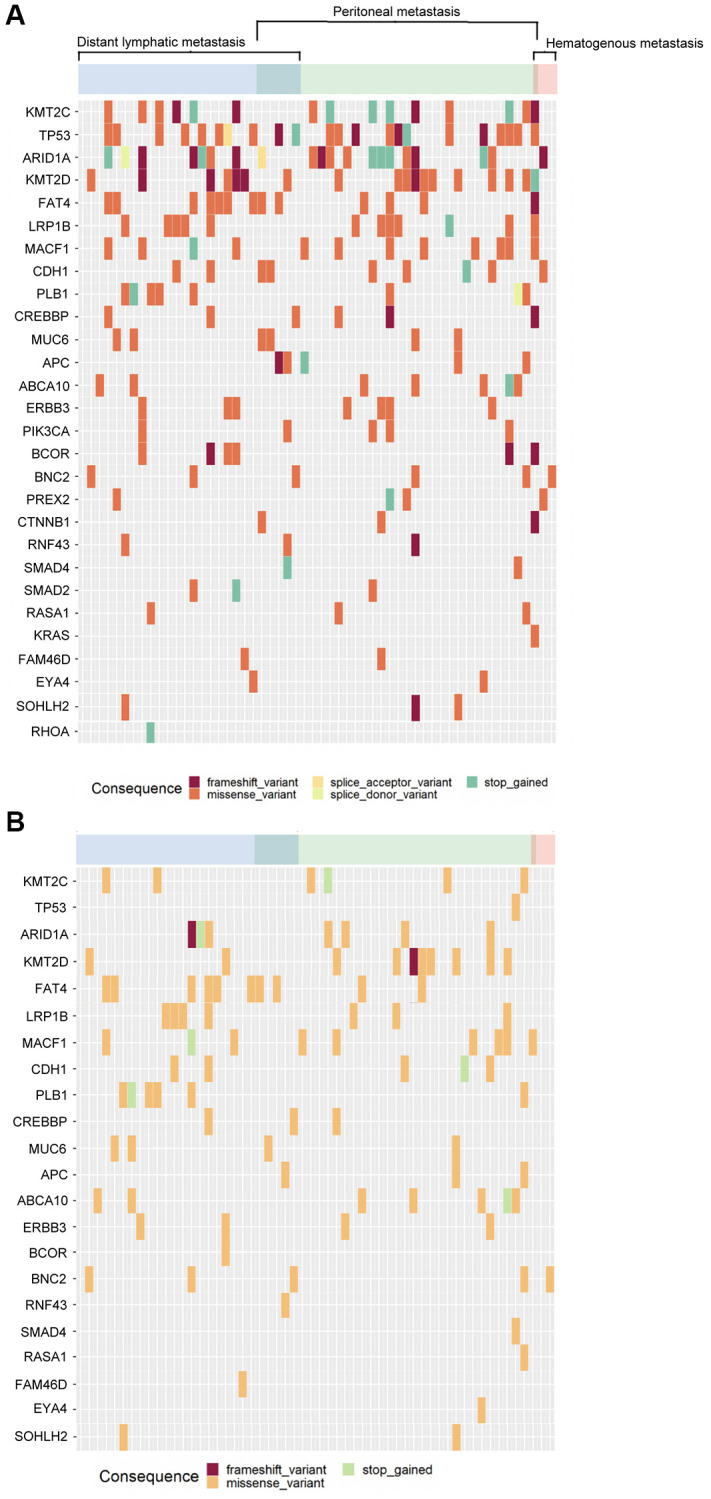
**The mutational patterns of the tumor tissue and cfDNA.** (**A**) Tumor tissue, (**B**) cfDNA.

In total, 621 mutation spots among these 29 genes were found in tumor samples. Among these 56 patients, 360 mutation spots in tumor samples in 45 patients could not be detected in cfDNA. The concordance of mutation spots in these 29 genes was 42.0% (261/621) between cfDNA and tumor samples. Figure 5 shows the concordance of mutation patterns in these 29 genes between tumor samples (Figure 5A) and cfDNA (Figure 5B). All the genetic mutations in cfDNA could be identified in tumor DNA.

### The mutation patterns of tumor DNA and cfDNA in patients with more than one metastatic pattern

Among the 56 stage IV GC patients, one patient had both peritoneal and hematogenous metastases. The patient had ten mutated genes in the tumor DNA and a total of 60 mutated spots, and only one mutated gene, MACF1, was detected in both tumor DNA and cfDNA.

Among the 56 stage IV GC patients, five patients had both peritoneal and distant lymphatic metastases. All of them had at least two mutated genes in tumor DNA and at least one mutated gene in cfDNA. Regarding tumor DNA, there were two mutated genes in one patient (*CDH1* and *MUC6*), five mutated genes in two patients (including *BNC2*, *FAT4*, *APC*, *TP53*, *CREBBP*), and ten mutated genes in two patients (*CDH1*, *PIK3CA*, *SMAD4*, *MUC6*, *FAT4*, *APC*, *RNF43*, *KMT2D*, *CTNNB1*, *ARID1A*). Regarding cfDNA, three patients had one mutated gene (including *MUC6* mutation in one patient and *FAT4* mutation in two patients), and two patients had two mutated genes (one patient with *BNC2* and *CREBBP* mutations; another patient with *RNF43* and *APC* mutations).

We further compared the number of mutated genes and mutated spots between single-site and multiple-site metastases in tumor DNA and cfDNA. Regarding tumor DNA, the number of mutated genes (3.5 ± 2.3 vs. 4.7 ± 2.9, *P* = 0.273) was not significantly different between single-site and multiple-site metastases, while patients with multiple-site metastases had significantly more mutated spots than patients with single-site metastases (23.7 ± 18.6 vs. 9.6 ± 8.6, *P* = 0.002). Regarding cfDNA, the number of mutated genes (1.8 ± 1.4 vs. 1.3 ± 0.5, *P* = 0.432) and mutated spots (5.0 ± 4.2 vs. 6.2 ± 2.3, *P* = 0.491) were not significantly different between single-site and multiple-site metastases.

## DISCUSSION

In the present study, NGS with a 29-gene panel was used to analyze cfDNA and tumor DNA in stage IV GC patients. The concordance of mutation patterns between cfDNA and tumor DNA was 42.0%. The specificity was 100% using the mutation status of cfDNA to predict mutation patterns in tumor samples. For cfDNA with *PLB1* mutations, patients were more likely to develop distant lymphatic metastasis than peritoneal metastasis. Patients with multiple-site metastases had significantly more mutated spots than patients with single-site metastases. To the best of our knowledge, this is the first study to investigate the mutation patterns of different metastatic patterns between cfDNA and tumor DNA for stage IV GC.

Our results showed that the concordance rate between tumor DNA and cfDNA was 42%, which was similar to previous studies [14]. Varkalaite et al., [14] used whole-exome sequencing for mutation analysis; among the 152 mutations in the plasma samples, only 39 mutations were detected in the tissue samples, indicating that the specificity was 25.7%. In our study, the specificity was 100% for cfDNA to predict mutations in tumor samples. The difference between our study and a previous study [14] might be due to different technology in mutation detection, differences in tumor stage and races. The quantity of cfDNA was higher in stage IV GC than in stage I–III GC [5], which might increase the detection rate of genetic mutations in cfDNA and make our results more promising. A recent published study [15] demonstrated that by using a 605-gene sequencing panel to sequencing cfDNA and tumor DNA, the mutation concordance between plasma cfDNA and tumor DNA was higher in stage IV GC (70.6%) than stage III GC (30.2%). cfDNA is useful in molecular profiling of GC patient and may be helpful in the prediction of patient survival, clinical therapeutic response, and future development of personalized therapy regimens. It was reported that the quantity of tissue matching alterations and presence of any somatic mutation in cfDNA is significant for discrimination between M0 and M1 GC patients, indicating that quantitative and qualitative cfDNA mutational profile analysis is useful for the evaluation of GC disease status and patient prognosis [14].

Anti-PD-L1 is approved as a primary immunotherapy in stage IV GC. Higher mutational load in cfDNA was associated with a better response to pembrolizumab, and reduced cfDNA six weeks after therapy had a longer progression-free survival (PFS), indicating that cfDNA can serve as a predictor of treatment response and PFS for GC [16]. Jin et al., reported that mutation status of *TGFBR2*, *RHOA*, and *PREX2* in cfDNA were associated with resistant to immunotherapy and a shorter PFS. Hence, cfDNA can serve as a biomarker in the response to immunotherapy in advanced GC [17]. It was reported that *CCNE1* amplification in cfDNA was associated with resistant to *HER2*-targeted therapy, while *HER2* amplification was sensitive to *HER2*-targeted therapy [18]. Consequently, cfDNA mutational profiles may serve as a potential biomarker in the prediction of immunotherapy and targeted therapy in GC. In addition, for stage I–III GC patients after curative surgery, monitoring cfDNA mutation patterns may serve as a liquid biopsy and a useful biomarker for predicting tumor recurrence, recurrence patterns and patient prognosis. For patients at a high risk of tumor recurrence according to the cfDNA mutation patterns, postoperative adjuvant therapy may be required for preventing tumor recurrence and improving patient survival. Our results may provide useful information regarding immunotherapy or targeted therapy for GC treatment in the future.

We also analyzed the correlation between metastatic pattern and genetic mutation pattern in tumor DNA and cfDNA in stage IV GC, which has not yet been reported. The four most commonly mutated genes among the three metastatic patterns were *TP53*, *KMT2C*, *KMT2D* and *ARID1A*, which were also among the top high-frequency mutations for GC reported by the Catalogue of Somatic Mutations in Cancer (COSMIC) database [19]. All four genes mentioned above could be considered tumor suppressor genes [20–23]. *TP53* mutation was associated with lymph node metastasis and distant metastasis in GC, especially in the Asian population [20], which was similar to our results. *ARID1A* mutation was reported to be associated with distant metastasis in GC [22], and *ARID1A* mutation could serve as a biomarker for immunotherapy in GI tract cancer [23]. *KMT2D* and *KMT2C* mutations in GC were associated with the DNA repair process, and these two genetic mutations were considered targets for cancer treatment using poly ADP-ribose polymerase (PARP) inhibitors [22, 24]. According to our results, some common genetic mutations were identified in different metastatic patterns, and drug therapy might be suitable for stage IV GC treatment, such as *PARP* inhibitors for mutations of the *KMT2* family and immunotherapy for *ARID1A* mutations.

Low expression of E-cadherin (CDH1), ARID1A, FAT4, and KMT2C were associated with a poor prognosis in GC [25–28], while high expression of p53 or KMT2D was reported to be associated with a poor survival [29, 30]. *MACF1* mutations were associated with upregulation of the mTOR signaling pathway and had a worse prognosis in breast cancer [31]. PLB1 (phospholipase B1) is a secreted membrane-associated phospholipase that is involved in choline metabolism in tumors [32]. *PLB1* mutation was reported to be associated with poor survival in non-small cell lung cancer and glioblastoma multiform [33, 34]. As shown in Table 2, our results demonstrated that the genetic mutations of these genes mentioned above (*CDH1*, *ARID1A*, *FAT4*, *KMT2C*, *TP53*, *KMT2D*, *MACF1*, and *PLB1*) were associated with the down regulation of the associated protein function, which may be involved in the up-regulation or down-regulation in the mechanism of cancer developing. In addition, our results showed that *PLB1* mutation was more common in the cfDNA of GC patients with single distant lymphatic metastasis than in patients with peritoneal metastasis, which has not yet been reported yet. According to our results, *PLB1* mutation in cfDNA may serve as a risk factor for distant lymphatic metastasis in GC.

Our results demonstrated that the sensitivity of 88.9% was the highest using cfDNA to predict the mutation patterns of tumor DNA with *FAT4* mutation. *FAT4* is involved in the Wnt pathway, which plays an important role in tumorigenesis, invasion, and vascularization [35]. *FAT4* silencing enhanced proliferation, colony formation, invasion, and metastasis and reduced fibronectin adhesion [36]. According to our results, monitoring of *FAT4* mutation in cfDNA may be a substitute for tumor biopsy in stage IV GC.

In colorectal cancer, higher cfDNA levels and increased number of genetic mutations in cfDNA were associated with a poor surgical and multiple-site metastasis [37], which has not yet been reported in GC. According to our results, GC patients with multiple-site metastases had at least two mutated genes in tumor DNA and at least one mutated gene in cfDNA. In tumor DNA, the number of mutated spots rather than the number of mutated genes was significantly higher in multiple-site metastases than in single-site metastases, which was not observed in cfDNA. It seems that more mutated spots in tumor DNA may diversify the mutation patterns and increase the possibility of multiple-site metastasis. Our findings may remind the physicians of the possibility of multiple-site metastasis when many mutated spots are found in tumor DNA.

There are limitations in the current study. First, this is a retrospective and single-center study. Second, the patient number was limited and selection bias may exist. More patients enrolled from different countries and races are required to verify our results.

## MATERIALS AND METHODS

### Patients and sample collection

Tumor and preoperative serum samples were collected from 56 stage IV GC patients from Taipei Veterans General Hospital Biobank. Only stage IV GC patients with available tumor and preoperative serum samples in the biobank were enrolled in this study. The exclusion criteria included patients who had stage I–III GC, who received emergent surgery, or who did not have available tumor or preoperative serum samples in the biobank. The tumor tissues and normal gastric mucosa tissues were collected and stored in a biobank at our institution. Written informed consent before tumor tissue collection was obtained from all study participants. The study was approved by the Institutional Review Board of Taipei Veterans General Hospital (2021-07-022AC). The pathological staging of GC was performed according to the 8th American Joint Committee on Cancer (AJCC)/Union for International Cancer Control (UICC) TNM classification system [38]. The metastatic patterns included peritoneal, hematogenous, and distant lymphatic metastases. Single-site metastasis was defined as a single metastatic pattern. Multiple-site metastasis was defined as a patient with more than one metastatic pattern.

### cfDNA extraction from plasma

Whole blood (4~8 ml) was drawn from each clinical patient using disposable venous blood lancet and stored directly in cfDNA Blood Collection Tube (Streck). Blood samples were stored at room temperature and used for plasma separation extraction within three days. In plasma separation, tube containing whole blood was centrifuged at 3,000 × g for 10 minutes at room temperature, then the upper layer was transferred to the 1.5 ml micro-centrifuge tube. The transferred tubes were processed for the second centrifuge at 11,000 × g for 10 minutes, and finally transfer the supernatant to new sample tubes for cfDNA extraction. cfDNA was extracted from 1,000 μl of plasma by using the QIAamp MinElute ccfDNA Kits (Qiagen), and tumor DNA was extracted from tissue specimens by using a QIAamp DNA Tissue Kit (Qiagen, Valencia, CA, USA). After DNA extraction, DNA quantity was measured by using the Qubit dsDNA High-Sensitivity assay (Thermo Fisher Scientific).

### Analysis of genetic alterations

A total of 250 ng DNA from each tumor tissue was used to construct an NGS library using an IDT Lotus Library Preparation Kit (IDT, USA). Each DNA sample was fragmented and then used to prepare a DNA library by performing end repair, a-overhang addition, adaptor ligation and size selection (250~350 bp). Fifteen microliters of each cfDNA sample was used to construct a library by using the xGen Prism DNA Library Prep Kit (IDT). Target DNA of exonic regions of 29 frequently-mutated genes in GC (*ABCA10*, *APC*, *ARID1A*, *BCOR*, *BNC2*, *CDH1*, *CNGA4*, *CREBBP*, *CTNNB1*, *ERBB3*, *EYA4*, *FAM46D*, *FAT4*, *KMT2C*, *KMT2D*, *KRAS*, *LRP1B*, *MACF1*, *MUC6*, *PIK3CA*, *PLB1*, *PREX2*, *RASA1*, *RHOA*, *RNF43*, *SMAD2*, *SMAD4*, *SOHLH2* and *TP53*) was enriched using probe-based methods. The probes were synthesized by Integrated DNA Technologies (USA) according to our previously designed probe sequences, and the capture procedure was performed following the IDT guidelines. After probe-based enrichment, libraries of each pool were amplified with 14 cycles. The amplified libraries were quantified using an LC480 qPCR system (Roche) and pooled into a new 1.5-ml tube as a 10-nM pooled DNA library. The final pool was used for sequencing (Illumina NextSeq sequencer, 2 × 150 bp). The raw output of each tumor tissue was >1.5 Gb, and the average depth of target regions was >250X. The raw output of each cfDNA was >15 Gb, and the average depth of target regions was >2000X. We used the Illumina Basespace Dragen somatic mutation pipeline (https://www.illumina.com/products/by-type/informatics-products/basespace-sequence-hub/apps/edico-genome-inc-dragen-somatic-pipeline.html) to perform variant calling and annotated all variants by using Illumina variant interpreter (https://variantinterpreter.informatics.illumina.com/home). In this study, although adjacent-normal parts or white blood cells were not collected for genotyping and identifying germline variants of each case, single-nucleotide polymorphisms, defined as the minor allele frequency >1%, of the primary variants of tumor and cfDNA were filtered out based on the databases of Taiwan biobank, 1,000 Genome and GnomAD East Asian populations. Variants with >5% and >1% frequencies were retained for the following in tissue and cfDNA, respectively. Although some variants were found to have high confidences in variant calling with more than 2,000 depth, however, only >1% variants were considered in this study, which might reduce the sensitivity in this study.

### Immunohistochemical (IHC) staining

IHC staining was performed according to the manufacturer’s recommendations, including E-cadherin (CDH1), MACF1, p53, PLB1, ARID1A, KMT2C, FAT4, and KMT2D. Antibodies utilized were: E-cadherin (Genemed #61-0192), MACF1 (Proteintech #13058-1-AP), p53 (Leica #P53-D07-L-CE), PLB1 (Invitrogen #PA5-119343), ARID1A (Sigma #HPA005456), KMT2C (Invitrogen #PA5-68419), FAT4 (Invitrogen #PA5-72970), KMT2D (Invitrogen #PA5-57490).

Immunoreactivity E-cadherin was localized in membranes; MACF1 and PLB1 were localized in the cytoplasm and membranes; p53 and ARID1A were localized in the nucleus; KMT2C and FAT4 were localized in the cytoplasm; KMT2D was localized in the cytoplasm and nucleus, and mainly in nucleus (Figure 2). Immunoreactivity of at least 10% of tumor cells was considered positive staining.

### Statistical analysis

IBM SPSS Statistics 25.0 was used for statistical analyses. A χ^2^ test with Yates correction or Fisher’s exact test was used to compare the categorical data. The frequency of the specific gene mutation was calculated using the number of patients carrying the specific gene mutation divided by the total number of the patients and was presented as a percentage. A *P* value < 0.05 was defined as statistically significant.

## CONCLUSIONS

Due to the high sensitivity and specificity of some genes in the prediction of mutation in tumor samples, monitoring the mutation pattern of cfDNA may be a substitute for tumor biopsy and may be applicable in the treatment of stage IV GC.
